# Rapid HPLC-DAD and Chemometric Discrimination of Raw Dark Tea from Three Specific Mountain Origins Within Anhua

**DOI:** 10.3390/foods15101664

**Published:** 2026-05-10

**Authors:** Wenyan Zeng, Chunlin Wu, Guiying Lu, Meng Dong, Xiaohong Zhou, Xiangdong Qing

**Affiliations:** 1 School of Materials and Chemical Engineering, Hunan City University, Yiyang 413000, China; 2 Yiyang Product Quality Supervision and Inspection Institute, Yiyang 413000, China; 3 Hunan Provincial Key Lab of Dark Tea and Jin-Hua, Hunan City University, Yiyang 413000, China

**Keywords:** HPLC-DAD, ATLD, t-SNE, flavan-3-ols, micro-terroir, Anhua dark tea

## Abstract

Anhua dark tea, a protected geographical indication product in Hunan, China, derives its value from specific mountain micro-terroirs, including Furongshan, Gaoma Erxi, and Yuntaishan. Across these areas, micro-terroir and cultivar variations impart distinctive chemical components to the decisive raw material (locally known as Hei Mao Cha). For the authentication of these specific origins, we developed a rapid HPLC-DAD method coupled with the ATLD algorithm, enabling the quantification of caffeine and seven major flavan-3-ols within five minutes. Our method achieved satisfactory accuracy, with average recoveries of 84.73–119.88% and RMSEP values ranging from 0.28 to 4.39 μg/mL. We subsequently applied PCA and FCA, which revealed distinct clustering patterns of the tea samples by their mountain origin. Notably, Furongshan and Gaoma Erxi exhibited markedly distinct chemical profiles, while Yuntaishan showed intermediate characteristics. This integrated HPLC-DAD/ATLD protocol, coupled with non-linear t-distributed stochastic neighbor embedding (t-SNE) followed by random forest (RF) classification (validation accuracy: 85.7%), offers a practical solution for the fine-scale geographical traceability of raw dark tea, supporting quality control and providing insights into how micro-terroir shapes tea chemistry. This approach can be readily adapted for the authentication of other geographical indication products.

## 1. Introduction

Anhua dark tea, a well-known post-fermented tea, holds protected geographical indication status in Hunan Province, China. The unique flavor and health benefits of Anhua dark tea, attributed to bioactive compounds like tea polyphenols and polysaccharides, have attracted considerable consumer interest [1,2,3,4]. In addition to its broad geographical designation, the premium quality and distinctive sensory characteristics of Anhua dark tea are closely associated with specific micro-terroirs within the county, particularly celebrated mountain origins including Furongshan, Gaoma Erxi, and Yuntaishan [5,6]. These adjacent areas exhibit subtle but influential variations in altitude, microclimate, soil, and tea cultivar composition. These micro-terroir factors impart characteristic chemical components to the raw dark tea (locally known as Hei Mao Cha), which serves as the decisive primary material governing the final product’s quality [7,8,9,10]. Therefore, authenticating the geographic origin of the raw dark tea at this initial processing stage is critical for protecting the geographical indication, ensuring product integrity, and scientifically elucidating terroir effects [11,12,13,14].

The chemical basis for geographical authentication of tea is well established. Chromatographic techniques, such as HPLC, LC-MS, and GC-MS, play a fundamental role in identifying and quantifying specific markers of origin [15,16,17,18,19,20]. Nevertheless, a major practical limitation is the time and cost required for comprehensive separation of complex tea matrices, which limits their utility for rapid, high-throughput traceability [21,22,23,24]. To address this, the integration of faster analytical methods with advanced chemometrics offers a powerful solution. HPLC-DAD provides a balance of accuracy and speed [25], while chemometric tools like second-order calibration and ATLD algorithm excel at resolving overlapping signals and extracting meaningful information from complex data [26,27]. This combined strategy has been successfully applied to related food authentication tasks, including the quality evaluation and authentication of various Chinese teas [28,29,30].

Despite these advancements, a significant gap persists in applying such a rapid, integrated strategy to the fine-scale geographical discrimination of raw dark tea at the primary processing stage. Specifically, distinguishing among these specific mountain origins within the same protected county remains a challenge. Filling this gap would provide a practical tool for supply chain traceability at its most critical point and offer novel insights into how micro-terroir shapes tea chemistry. Consequently, this study aimed to develop and validate a rapid HPLC-DAD method coupled with comprehensive chemometric analysis to accurately discriminate Anhua raw dark tea samples from three specific mountain origins: Furongshan, Gaoma Erxi, and Yuntaishan.

## 2. Materials and Methods

### 2.1. Chemicals

All reagents and chemicals were of analytical or HPLC grade, obtained from commercial sources, and used as received. Ultrapure water was prepared using a Milli-Q system (Millipore, Burlington, MA, USA). Eight reference standards, comprising seven flavan-3-ols (GC, EGC, GCG, CG, EC, EGCG, ECG) and caffeine (CAF), were obtained from Shanghai Yuanye Biotechnology Co., Ltd (Shanghai, China). The purity of all standards was ≥98%. HPLC-grade methanol (≥99.99%) was supplied by Tiandi Environmental Protection Co., Ltd (Shanghai, China). Acetic acid and acetonitrile (HPLC grade, ≥99.99%) were supplied by Shanghai Dowian Reagent Co., Ltd (Shanghai, China).

### 2.2. Sample Collection and Preparation

A total of 63 raw dark tea samples were collected during the summer production season of 2023. The samples originated from three specific mountain areas within Anhua County, Hunan Province, China: Yuntaishan (n = 23), Gaoma Erxi (n = 18), and Furongshan (n = 22). All samples were obtained directly from local primary processing workshops within their respective production areas to ensure authenticity. Immediately after collection, the tea samples were ground into a fine powder, using a FW-100 high-speed universal grinder (60-mesh sieve). The powder was sealed and stored at −20 °C until further analysis.

For extraction, a mixed solvent was first prepared by combining 25 mL of ascorbic acid solution, 25 mL of Na_2_EDTA solution, and 50 mL of acetonitrile, then diluting the mixture to a final volume of 500 mL with distilled water. Ascorbic acid was added to the extraction solvent to prevent oxidation of phenolic compounds (flavan-3-ols and caffeine) during the extraction process. The resulting solution was then mixed with 99% methanol in a volume ratio of 3:7 to obtain the extract. A total of 0.2 g of tea sample powder was weighed, and 4 mL of the extraction solution was added. It was then thoroughly mixed, sonicated in water at 70 °C for 10 min, and centrifuged at 5000 rpm for 5 min. One milliliter of the supernatant was removed and transferred to a 10-mL brown volumetric flask. The volume was then adjusted with the extract, and the solution was filtered through a 0.45 μm Polyvinylidene Fluoride (PVDF) syringe filter before being placed in a liquid chromatography injection vial. Voucher specimens were deposited at the Herbarium of Hunan City University (accession numbers HCU-2023-001 to HCU-2023-063).

### 2.3. Preparation of Standard Solutions

Methanol was used as the solvent to prepare individual stock solutions of the eight standard substances (GC, EGC, EC, EGCG, CAF, ECG, CG, and GCG), each at a concentration of 0.2 mg/mL. These stock solutions were stored at 4 °C in the dark when not in use. To validate the method, a calibration set consisting of seven samples (labeled J01–J07) and a validation set of three samples (labeled Y01–Y03) were prepared by appropriately mixing and diluting the stock solutions. The specific concentration of each analyte in these samples is detailed in Table 1.

### 2.4. Instrumentation

The analytical balance (model TP-214) was a precision analytical balance with a sensitivity of 0.0001 g from Sartorius Instrument Co., Ltd. (Beijing, China). A TD4C centrifuge from Shanghai Photosynthesis Co., Ltd. (Shanghai, China) was used. The ultrasonic oscillator (model SHZ-D (III)) was obtained from Shanghai Yushen Instrument Co., Ltd. (Shanghai, China).

An HPLC system (Shimadzu LC-20AT, Kyoto, Japan) was used, consisting of a degassing unit, two LC-20AD binary pumps, an SIL-20A autosampler, a CTO-20A column oven, and an SPD-M20A DAD equipped with a deuterium lamp. The DAD operated across a wavelength range of 190–800 nm with a 1.2 nm interval. The DAD was set to 270 nm for quantification and scanned from 190–400 nm for spectral identification. Compounds were identified by comparing retention times and UV spectra with authentic standards. Chromatographic separation was carried out on a Shim-pack GIST C18 reversed-phase column (250 mm × 4.6 mm i.d., 5 μm; Shimadzu, Kyoto, Japan). Isocratic elution was performed at a flow rate of 1.0 mL/min, with an injection volume of 20 μL. Data acquisition and processing were carried out using the instrument’s LabSolution software (Version 5.90). Prior to injection, all sample solutions were filtered through 0.45 μm disposable non-sterilized PVDF syringe filters.

### 2.5. Chromatographic Conditions

Chromatographic separation was performed under isocratic conditions. The mobile phase consisted of 0.1% (*v*/*v*) formic acid in water (solvent A) and 0.1% (*v*/*v*) formic acid in methanol (solvent B), delivered at a constant flow rate of 1.0 mL/min with a composition of A:B = 55:45 (*v*/*v*). The column temperature was maintained at 35 °C, and the injection volume was 20 μL. Under these conditions, the system backpressure remained stable at approximately 11.3–11.4 MPa.

### 2.6. Data Processing with ATLD Algorithm

The chromatographic data from raw dark tea samples, acquired via the HPLC-DAD system, were initially processed using the instrument’s built-in LabSolution software and then exported. To resolve overlapping peaks and mitigate matrix effects, the algorithm alternating trilinear decomposition (ATLD) was applied. This algorithm decomposes the three-way data array **X** (with frontal slices to obtain the pure chromatographic (**A**), spectral (**B**), and relative concentration (**C**) profiles. Its robustness to high collinearity and unknown interferences enables accurate quantification [31,32].

The three-way array X was constructed with dimensions of 63 samples × 400 elution time points × 150 wavelengths (190–400 nm). Four specific elution zones (2.9227–3.0613, 3.0720–3.7013, 3.7120–4.0213, and 4.0320–4.6827 min) were selected based on visual inspection of standard chromatograms and assessment of peak overlap. The number of components for each zone was determined by core consistency diagnostics, and the convergence criterion was set to 1 × 10^−6^.

### 2.7. Unsupervised Chemometric Analysis (PCA and FCA)

ATLD and principal component analysis (PCA) were implemented in the MATLAB computing environment (Version R2015b, MathWorks Inc., Natick, MA, USA). Subsequently, feature component analysis (FCA) and all graphical representations (including charts, chromatograms, and three-dimensional maps) were performed and generated using SigmaPlot software (Version 14.0, Systat Software Inc., San Jose, CA, USA, www.systatsoftware.com). All computations were performed on a computer running the Microsoft Windows 11 operating system. PCA and FCA are unsupervised methods used for dimensionality reduction and trend visualization, not for formal classification.

### 2.8. Supervised Classification (LDA, PLS-DA, and t-SNE-RF)

For supervised classification, both linear and non-linear approaches were evaluated. Linear discriminant analysis (LDA) and partial least squares discriminant analysis (PLS-DA) were performed. Given the complex nature of metabolomic data, t-distributed stochastic neighbor embedding (t-SNE) combined with random forest (RF) was additionally employed.

For PLS-DA, five-fold cross-validation was used to determine the optimal number of latent variables. For t-SNE-RF, hyperparameters were optimized using an orthogonal experimental design L9 (3^4^), evaluating perplexity (5, 20, 50), learning rate (10, 100, 1000), iterations (250, 500, 1000), and early exaggeration (4, 8, 12). The optimal configuration was determined by validation accuracy. t-SNE used the “correlation” distance metric. The RF model consisted of 500 trees and used Gini impurity for node splitting. Samples were randomly divided into training (n = 56, 88.9%) and validation (n = 7, 11.1%) sets. The detailed hyperparameter optimization results for t-SNE are provided in Table S4 (Supplementary Material).

## 3. Results

### 3.1. Development of a Rapid HPLC-DAD Method

**Figure 1:**
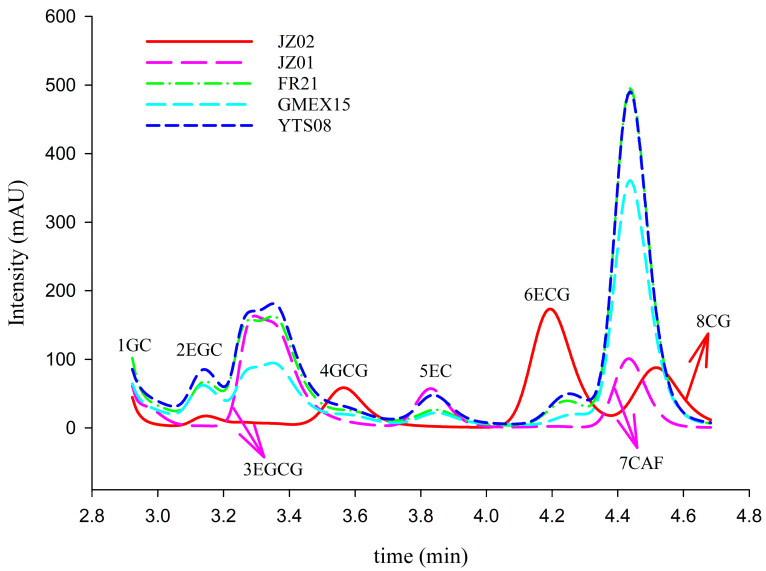
HPLC-DAD chromatograms of calibration samples (Jz01, Jz02) and authentic tea samples from Furongshan (FR21), Gaoma Erxi (GMEX15), and Yuntaishan (YTS08). HPLC conditions: mobile phase A (0.1% formic acid in water) and B (0.1% formic acid in methanol) with an isocratic elution of A:B = 55:45 (*v*/*v*); wavelength 270 nm; flow rate 1.0 mL/min; injection volume 20 μL; column temperature 35 °C. Peaks: 1. GC; 2. EGC; 3. EGCG; 4. GCG; 5. EC; 6. ECG; 7. CAF; 8. CG.

A rapid HPLC-DAD method was established for the quantification of caffeine and seven flavan-3-ols in raw dark tea. After systematic optimization of mobile phase composition, elution mode, and column temperature, optimal separation was achieved using an isocratic elution of 0.1% formic acid in water and methanol (55:45, *v*/*v*) at 35 °C with a flow rate of 1.0 mL/min. Figure 1 illustrates the complete baseline separation of all eight target analytes within 5 min using this method, laying the groundwork for high-throughput analysis. Linear calibration curves were constructed across concentration ranges of 6–200 μg/mL for GC to 40–200 μg/mL for ECG, demonstrating good linearity for all compounds.

### 3.2. Method Validation Using ATLD Algorithm

The analytical performance of the developed method was rigorously validated using a second-order calibration approach with the ATLD algorithm, following the International Council for Harmonisation (ICH) Q2 (R1) guidelines for recovery, precision (RMSEP), linearity, LOD, and LOQ. Excellent quantitative results were obtained from the analysis of seven calibration and three prediction samples (Table 2). High accuracy was demonstrated by average recoveries ranging from 84.73% to 119.88%. Notably, 22 of the 24 individual recovery measurements fell within the acceptable 90–108% range. The two outliers (CG 170.73% and ECG 60.95% in Y01) likely arise from matrix effects at concentration levels approaching the limit of quantification. Precision was confirmed by low root mean square error of prediction (RMSEP) values (0.28–4.39 μg/mL). The limits of detection (LOD, 0.42–8.58 μg/mL) and quantification (LOQ, 0.41–2.83 μg/mL) were suitable for tea sample analysis, validating the method’s sensitivity and reliability. These results confirm the robustness and accuracy of the ATLD-based quantification strategy.

### 3.3. Chemical Profiling of 63 Raw Dark Tea Samples

**Figure 2:**
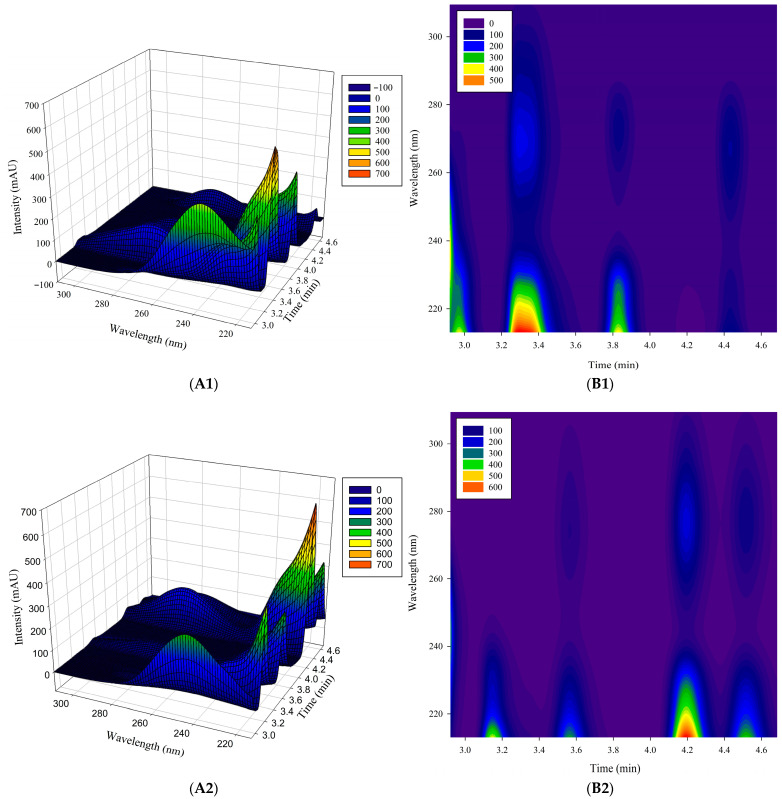

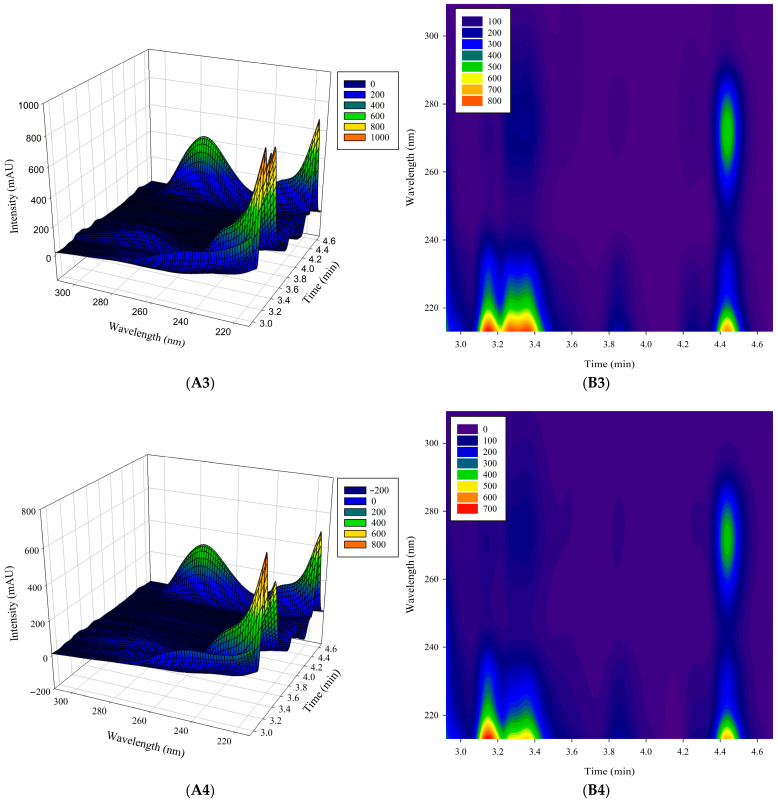

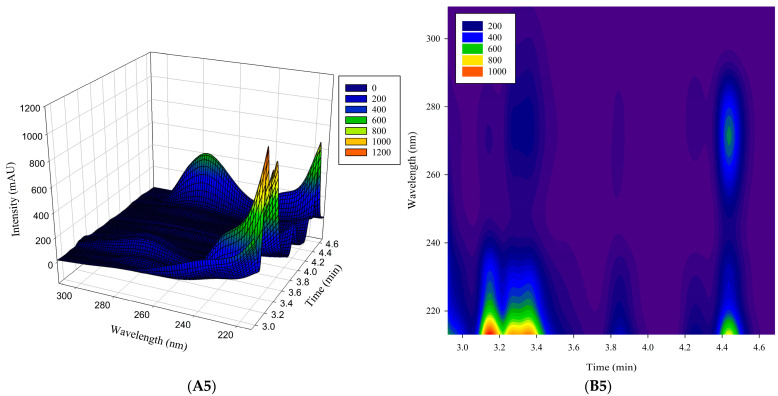
Representative three-dimensional chromatograms (**A1**–**A5**) and corresponding contour plots (**B1**–**B5**) of calibration samples Jz01 and Jz02 and authentic tea samples FR21 (Furongshan), GMEX15 (Gaoma Erxi), and YTS08 (Yuntaishan).

**Figure 3:**
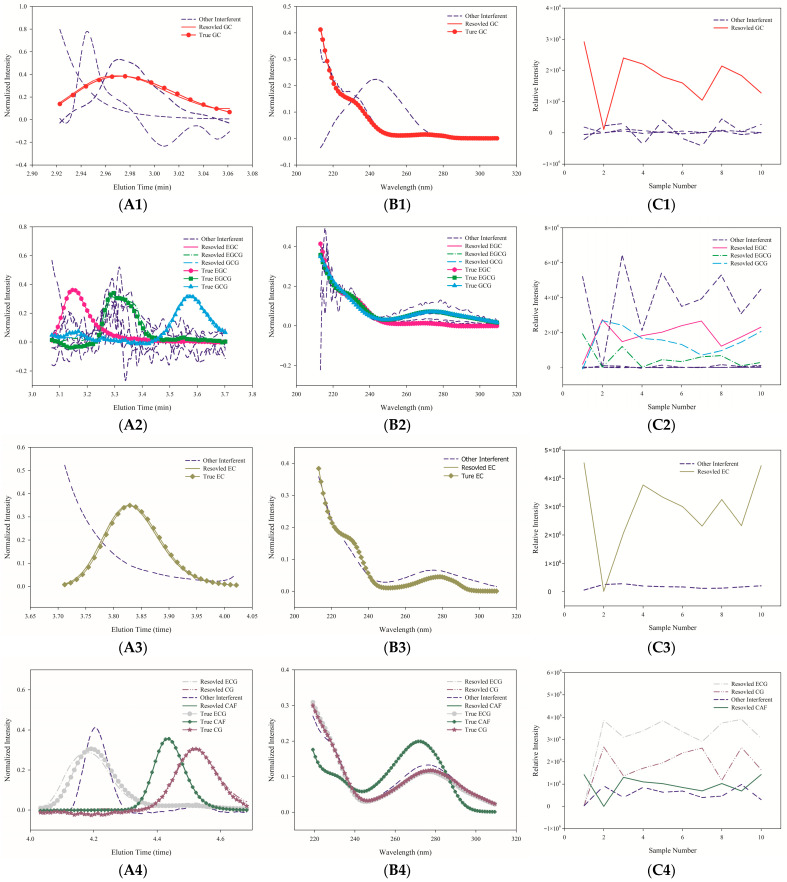
Resolution of overlapping peaks in four elution sub-regions using the ATLD algorithm. Left panels (**A1**–**A4**): normalized chromatographic profiles; Middle panels (**B1**–**B4**): normalized spectral profiles; Right panels (**C1**–**C4**): relative concentration profiles. Sub-region time windows: 1 (2.9227–3.0613 min), 2 (3.0720–3.7013 min), 3 (3.7120–4.0213 min), and 4 (4.0320–4.6827 min).

The validated method was applied to analyze 63 raw dark tea samples from three mountain origins: Furongshan (n = 22), Gaoma Erxi (n = 18), and Yuntaishan (n = 23). The complex matrix of the tea samples, depicted by representative three-dimensional chromatograms and contour plots in Figure 2, presented significant peak overlap. This issue was addressed by applying the ATLD algorithm to four specific elution zones (2.9227–3.0613, 3.0720–3.7013, 3.7120–4.0213, and 4.0320–4.6827 min), which successfully resolved the chromatographic and spectral profiles, as demonstrated in Figure 3. The resolved spectra showed high correlation with authentic standards (correlation coefficients: 0.8350–0.9989), confirming the fidelity of mathematical separation.

The complete concentration data for all 63 individual raw dark tea samples from Furongshan (n = 22), Gaoma Erxi (n = 18), and Yuntaishan (n = 23) are provided in Tables S1–S3 of the Supplementary Material. Table 3 below summarizes the mean concentration and standard deviation of each analyte for each origin. Notably, some samples showed extreme values: GMEX06 had an exceptionally low total concentration (14.78 μg/mL), while FR07 had the highest EGCG level (198.22 μg/mL).

As shown in Table 3, caffeine and EGCG were the most abundant compounds across all three origins, while GCG was consistently below the detection limit in all samples. Notably, substantial within-origin variability was observed, particularly for EGCG (coefficients of variation ranging from 76% to 101%), reflecting the inherent heterogeneity of tea materials from different micro-terroirs and tea cultivars within each mountain area. Among the three origins, Yuntaishan samples exhibited the highest mean EGCG concentration (64.76 μg/mL), followed by Furongshan (57.31 μg/mL) and Gaoma Erxi (34.80 μg/mL), whereas Gaoma Erxi showed the highest mean EC and EGC levels.

### 3.4. Unsupervised Pattern Recognition (PCA and FCA)

**Figure 4:**
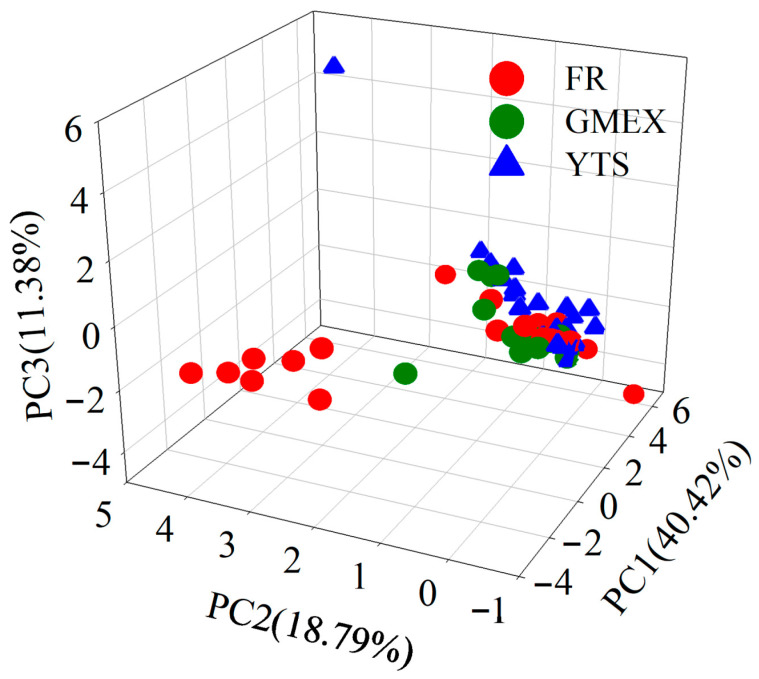
PCA score plot showing clustering tendencies of raw dark tea samples from three mountain origins.

**Figure 5:**
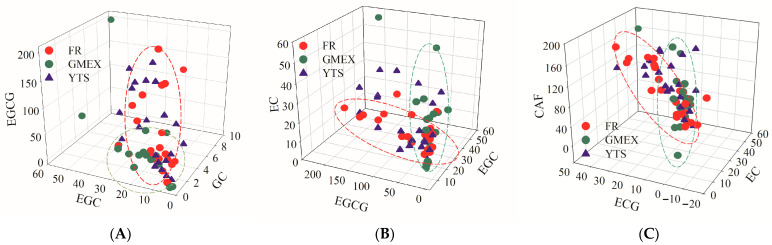
FCA for identifying key discriminatory markers. FCA plots based on the relative concentrations of key analyte triads: (**A**) EGCG, EGC, and GC; (**B**) EC, EGCG, and EGC; (**C**) CAF, ECG, and EC.

Chemometric analysis revealed clear clustering tendencies of the samples by geographical origin. PCA revealed clear clustering patterns, as detailed in Figure 4: samples from Furongshan and Gaoma Erxi formed two distinct, well-separated groups, while those from Yuntaishan exhibited a more dispersed distribution with partial overlap with both other groups. FCA identified specific flavan-3-ol ratios and caffeine content as key discriminatory variables, with the corresponding results presented in Figure 5. Combinations such as EGC/GC/EGCG and ECG/EC/CAF were particularly effective in distinguishing the origins.

### 3.5. Supervised Classification Results

Three supervised classification methods were evaluated on a validation set of 7 independent samples (Furongshan: 2, Gaoma Erxi: 2, Yuntaishan: 3). The classification results are summarized in Table 4.

PLS-DA with five-fold cross-validation selected 8 latent variables. Variable Importance in Projection (VIP) scores identified EGCG (1.84), CAF (1.56), and EC (1.41) as the most discriminative compounds. The suboptimal performance of linear classifiers (LDA: 42.9%; PLS-DA: 57.1%) suggests that the chemical differences among the three mountain origins are not linearly separable in the original feature space.

Given the limited performance of linear classifiers, a non-linear approach combining t-SNE with RF was developed. Hyperparameters were systematically optimized using an orthogonal experimental design L9 (3^4^) (Table S4). The optimal configuration was achieved with perplexity = 50, learning rate = 100, iterations = 250, and early exaggeration = 12, using the “correlation” distance metric.

**Figure 6:**
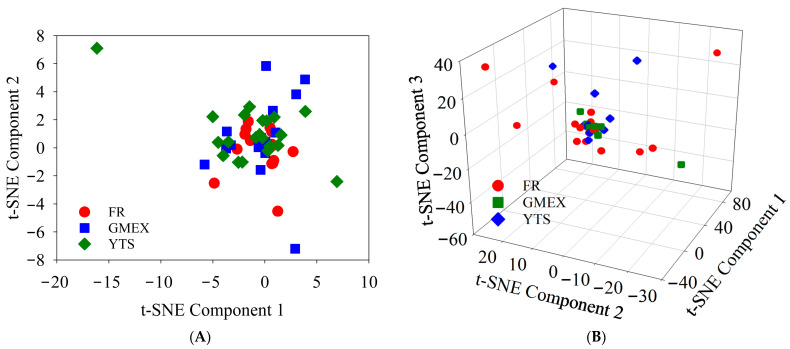
t-SNE visualization of raw dark tea samples from three mountain origins (perplexity = 50, learning rate = 100, iterations = 250). (**A**) Two-dimensional projection; (**B**) 3D projection.

Under the optimal configuration, the t-SNE-RF model achieved a training accuracy of 86.0% and a validation accuracy of 85.7% (6 out of 7 validation samples correctly classified). The confusion matrix showed that all three Gaoma Erxi validation samples were correctly identified, with only one Yuntaishan sample misclassified as Furongshan. The silhouette coefficient was 0.82 for the 3D projection, indicating well-separated clusters. Figure 6 shows the 2D and 3D t-SNE visualization of the training set classification results.

The superior performance of the non-linear t-SNE-RF model compared to linear classifiers (LDA: 42.9%; PLS-DA: 57.1%) demonstrates that micro-terroir chemical components are inherently non-linear, necessitating non-linear approaches for accurate geographical authentication.

The t-SNE visualization (Figure 6) reveals distinct clustering patterns among the three origins. In the 2D projection (Figure 6A), GMEX samples (green) form a relatively tight cluster, indicating good chemical homogeneity within this origin. FR samples (red) exhibit slightly more dispersion, reflecting greater within-origin compositional variability. YTS samples (blue) are positioned between FR and GMEX, with partial overlap toward the FR cluster, consistent with their intermediate chemical characteristics observed in PCA. One YTS sample (indicated by an arrow in Figure 6A) lies in proximity to the FR cluster, corresponding to its misclassification as FR in the confusion matrix (Table 4). The 3D projection (Figure 6B) achieves better separation of the three groups, with a silhouette coefficient of 0.82 confirming well-separated clusters.

## 4. Discussion

This study successfully tackled the challenge of fine-scale geographical traceability for Anhua raw dark tea by employing an integrated analytical strategy that combines rapid HPLC-DAD with advanced chemometrics. The efficient separation of eight target analytes within 5 min marks a substantial improvement over conventional methods, which generally require 30 min or longer [33]. This advancement enables practical, high-throughput screening essential for quality control applications.

The ATLD algorithm was crucial for accurate quantification within complex tea matrices. By leveraging the “second-order advantage,” this approach effectively resolved overlapping peaks and mitigated interference from uncalibrated components [34,35,36,37], overcoming limitations of traditional univariate methods. The high correlation between resolved and actual spectra (0.8350–0.9989) and satisfactory validation parameters confirm the reliability of this mathematical separation strategy. This combination of “mathematical separation” with optimized “physical separation” represents an efficient approach for analyzing complex natural products [38,39].

The chemometric results provide compelling evidence for the influence of micro-terroir on tea chemistry. The clear separation between Furongshan and Gaoma Erxi samples in PCA space aligns with their distinct geographical and environmental conditions, suggesting that altitude, microclimate, soil composition, and potentially cultivar differences impart characteristic chemical components to the raw material [40]. This finding validates the empirical recognition of “mountain-top” characteristics and underscores the importance of origin authentication at the raw material stage for protecting geographical indication products.

The intermediate position of the Yuntaishan samples presents an interesting case. This pattern may reflect several factors: heterogeneous growing conditions within Yuntaishan leading to greater compositional variability; cultivation of tea cultivars sharing chemical traits with both other regions; or environmental features placing Yuntaishan in a transitional zone. However, confirming these hypotheses requires additional data from soil analysis, cultivar genotyping, and multi-year sampling [41].

The identification of specific flavan-3-ol ratios and caffeine content as discriminatory markers through FCA provides actionable insights for quality control. These chemical indicators, particularly the relative abundances of EGCG, EGC, EC, and caffeine, offer measurable parameters for origin verification that could be implemented in practical screening protocols. The PLS-DA results showed moderate classification performance, with a cross-validation accuracy of 89.5% on the training set, though the independent validation accuracy was 57.1%, highlighting the need for non-linear approaches such as t-SNE-RF. The consistency between VIP scores and FCA results strengthens the reliability of the identified markers. The absence of an internal standard is a limitation of this study. While the ATLD algorithm and recovery experiments partially compensate for matrix effects and injection variability, the quantitative results, particularly for compounds with recovery values at the extremes (e.g., CG and ECG in sample Y01), should be interpreted with caution. Future work should incorporate an appropriate internal standard to further improve quantitative accuracy and robustness.

Compared with conventional HPLC methods for tea analysis, our approach offers several advantages. Traditional methods typically require 30–60 min for chromatographic separation [34], whereas our method achieves baseline separation of eight analytes within 5 min. The integration of the ATLD algorithm effectively resolves co-eluting peaks without exhaustive optimization of mobile phase composition. This combination of rapid physical separation and mathematical resolution provides a practical solution for high-throughput quality control applications. Similar chemometric-assisted strategies have been successfully applied to other tea authentication tasks [38], supporting the generalizability of our approach.

An important methodological finding is the substantial performance difference between linear (LDA, PLS-DA) and non-linear (t-SNE-RF) classifiers. Linear methods yielded validation accuracies of 42.9–57.1%, while the non-linear approach achieved 85.7% accuracy. This discrepancy likely reflects the inherent non-linearity of metabolomic data arising from micro-terroir variations. Environmental factors such as altitude, soil composition, and microclimate do not exert linear, additive effects on tea secondary metabolism; rather, they interact in complex, synergistic ways. These findings align with recent studies demonstrating that non-linear machine learning methods often outperform linear methods in food authentication tasks involving complex, multi-factorial environmental influences. For practical traceability applications, this study recommends the adoption of non-linear classification strategies when authenticating narrowly defined geographical origins within a small production region.

## 5. Conclusions

In this study, an efficient analytical strategy integrating rapid HPLC-DAD with chemometric methods was developed and validated for discriminating Anhua raw dark tea from three specific mountain origins. The optimized chromatographic conditions allowed for the separation of eight bioactive compounds within 5 min, while the ATLD algorithm ensured accurate quantification despite complex matrix interference. Method validation confirmed excellent performance, with recoveries ranging from 84.73% to 119.88% and RMSEP values between 0.28 and 4.39 μg/mL.

The application of this approach to 63 samples from Furongshan, Gaoma Erxi, and Yuntaishan revealed distinct chemical profiles associated with each origin. PCA and FCA effectively visualized the grouping patterns of the samples, with Furongshan and Gaoma Erxi showing clear separation and Yuntaishan exhibiting intermediate characteristics. Supervised classification using t-SNE combined with random forest achieved a validation accuracy of 85.7%, substantially outperforming linear classifiers (LDA: 42.9%; PLS-DA: 57.1%). This finding highlights the non-linear nature of micro-terroir-driven chemical variations and underscores the value of non-linear machine learning approaches for geographical authentication tasks. These findings demonstrate that subtle micro-terroir variations within a small geographical area impart measurable chemical differences to raw dark tea, which aligns with previous studies on the influence of altitude and microclimate on tea chemistry [11,13].

The proposed methodology offers significant advantages for tea authentication: (1) rapid analysis suitable for high-throughput screening, (2) mathematical resolution of complex signals enabling accurate quantification, and (3) effective visualization of geographical patterns through multivariate analysis. This integrated approach provides a practical and reliable tool for quality control, geographical indication protection, and scientific investigation of terroir effects in dark tea production. A limitation of this study is the lack of an independent external validation set. Therefore, future work should assess model robustness using samples from different harvest years and locations and further investigate the relationship between raw material chemistry and final tea product characteristics. Furthermore, integrating advanced techniques such as metabolomics or isotopic analysis could provide deeper insights into the terroir-driven chemical variation in raw dark tea.

## Figures and Tables

**Table 1:** Concentrations of calibration (J01–J07) and validation (Y01–Y03) samples (μg/mL).

Sample	GC	EGC	GCG	CG	EC	EGCG	ECG	CAF
J01	20.00	0.00	0.00	0.00	40.00	93.10	0.00	31.60
J02	0.00	20.00	20.00	40.00	0.00	0.00	60.00	0.00
J03	18.00	10.00	18.00	20.00	38.00	83.89	40.00	28.44
J04	14.00	12.00	14.00	26.00	34.00	27.91	56.00	25.28
J05	12.00	14.00	12.00	30.00	30.00	65.17	52.00	22.12
J06	10.00	16.00	10.00	34.00	26.00	46.55	48.00	18.96
J07	6.00	18.00	6.00	38.00	20.00	55.86	40.00	15.80
Y01	16.00	8.00	8.00	20.00	30.00	74.48	50.00	22.12
Y02	12.00	12.00	12.00	40.00	20.00	37.24	60.00	15.80
Y03	8.00	16.00	16.00	28.00	40.00	55.86	40.00	31.60

**Table 2:** Quantitative and statistical results of the validation set were analyzed using ATLD.

Sample	Predicted Concentration (μg/mL) [Recovery (%)]							
	GC	EGC	GCG	CG	EC	EGCG	ECG	CAF
Y01	14.50 [90.61]	7.73 [96.58]	7.75 [96.86]	34.15 [170.73]	31.10 [103.67]	73.90 [99.22]	30.48 [60.95]	22.81 [103.14]
Y02	12.24 [102.01]	11.72 [97.68]	11.27 [93.94]	35.61 [89.03]	23.75 [118.77]	42.98 [115.43]	57.22 [95.36]	15.45 [97.78]
Y03	8.17 [102.14]	15.93 [99.56]	15.73 [98.30]	27.97 [99.88]	40.50 [101.25]	62.55 [111.97]	39.15 [97.87]	31.70 [100.31]
^1^ AVE	98.25	97.94	96.37	119.88	107.89	108.87	84.73	100.41
^2^ RMSEP	1.0825	0.2806	0.5769	4.3895	2.7882	6.2463	2.9113	0.5540
^3^ DEV	0.0509	0.0108	0.0162	0.0543	0.0725	0.0643	0.0125	0.0182
^4^ R	0.9926	0.9970	0.9971	0.8635	0.8433	0.9518	0.8350	0.9989
^5^ LOQ	2.3881	1.4980	0.3856	4.4564	0.2606	28.0628	8.5824	2.9394
^6^ LOD	0.7881	0.4943	0.1272	1.4706	0.0860	9.2607	2.8322	0.9700

^1^. AVE: average recovery rate of the three validation samples (Y01–Y03) (%).The remaining 22 individual recovery measurements all fell within the acceptable 90–108% range. ^2^. RMSEP: root mean square error of prediction (μg/mL), calculated as $RMSEP=[\frac{1}{I-1}\sum (c_{act}-c_{pred}{)}^{2}{]}^{1/2}$, where I is the number of prediction samples. ^3^. DEV: positive and negative deviation between predicted and nominal concentrations. ^4^. R correlation coefficient between predicted and nominal concentrations. ^5^. LOQ: limit of quantification (μg/mL). ^6^. LOD: limit of detection (μg/mL).

**Table 3:** Summary of predicted concentrations (mean ± SD ^1^, μg/mL) for eight analytes in raw dark tea from three mountain origins.

Origin	n	GC	EGC	EGCG ^2^	GCG ^3^	EC	ECG	CAF	CG
Furongshan	22	2.78 ± 1.54	15.82 ± 6.62	57.31 ± 57.99	0	19.19 ± 3.99	10.48 ± 12.24	105.58 ± 38.69	0.17 ± 0.39
Gaoma Erxi	18	2.03 ± 2.14	21.74 ± 14.26	34.80 ± 45.91	0	24.24 ± 11.73	5.80 ± 11.28	92.24 ± 41.50	0.90 ± 1.84
Yuntaishan	23	2.83 ± 1.36	17.63 ± 10.33	64.76 ± 55.88	0	24.11 ± 7.57	10.79 ± 11.34	116.95 ± 31.67	0.53 ± 0.60

^1^. Values are presented as mean ± standard deviation (SD). ^2^. The relatively large SD for EGCG indicates substantial within-origin compositional variation. ^3^.GCG was below the limit of detection (LOD) in all samples from all three origins.

**Table 4:** Confusion matrices and validation accuracies of supervised classification methods *.

Method	Confusion Matrix				Classification Accuracy
	Actual	FR	GMEX	YTS	
LDA	FR	1	2	1	42.9% (3/7)
	GMEX	0	2	0	
	YTS	0	1	0	
PLS-DA	FR	2	0	0	57.1% (4/7)
	GMEX	0	0	2	
	YTS	1	0	2	
t-SNE-RF	FR	1	0	0	85.7% (6/7)
	GMEX	0	3	0	
	YTS	1	0	2	

* Note: Rows and columns represent FR, GMEX, and YTS in order (0 = FR, 1 = GMEX, 2 = YTS).

## Data Availability

The data presented in this study are available upon request from the corresponding authors.

## Supplementary Materials

The following supporting information can be downloaded at https://www.mdpi.com/article/10.3390/foods15101664/s1, Table S1: Predicted concentrations of eight standard substances in 21 samples from Furongshan (μg/mL); Table S2: Predicted concentrations of eight standard substances in 18 samples from Gaoma Erxi (μg/mL); Table S3: Predicted concentrations of eight standard substances in 23 samples from Yuntaishan (μg/mL); Table S4: Orthogonal experimental design L9 (3^4^) for t-SNE hyperparameter optimization.

## Abbreviations

The following abbreviations are used in this manuscript:

AR	Average recovery
ATLD	Alternating Trilinear Decomposition
CAF	Caffeine
CG	Catechin gallate
DEV	Deviation
EC	Epicatechin
ECG	Epicatechin gallate
EGC	Epigallocatechin
EGCG	Epigallocatechin gallate
FCA	Feature Component Analysis
GC	Gallocatechin
GCG	Gallocatechin gallate
HPLC-DAD	High-performance liquid chromatography with diode-array detection
ICH	International Council for Harmonisation
LDA	Linear Discriminant Analysis
LOD	Limit of Detection
LOQ	Limit of Quantification
PCA	Principal Component Analysis
PLS-DA	Partial Least Squares Discriminant Analysis
PVDF	Polyvinylidene Fluoride
R	Correlation coefficient
RF	Random Forest
RMSEP	Root Mean Square Error of Prediction
t-SNE	t-distributed Stochastic Neighbor Embedding
VIP	Variable Importance in Projection
