# Supplementary material for: Rapid HPLC-DAD and Chemometric Discrimination of Raw Dark Tea from Three Specific Mountain Origins Within Anhua
Source: Foods. 2026 May 10;15(10):1664. doi: 10.3390/foods15101664 (PMC13206714; doi:10.3390/foods15101664)
Supplement: Supplementary file 1 [file foods-15-01664-s001.zip › foods-4237271-supplementary.pdf]

Supporting Information for

Rapid HPLC-DAD and Chemometric Discrimination of Raw Dark Tea from Three Specific Mountain Origins in Anhua

Wenyan Zeng, Chunlin Wu, Guiying Lu, Meng Dong, Xiaohong Zhou and Xiangdong Qing

Table S1. Predicted concentrations of eight standard substances in 21 samples from Furongshan (μg/mL).

Samples from Furong Mountain.	predicted values (μg/mL)							
	GC	EGC	EGCG	GCG	EC	ECG	CAF	CG
FR01	2.59	13.02	65.48	0	17.06	15.99	107.16	0
FR02	3.05	23.13	123.30	0	20.72	16.62	163.01	0
FR03	3.11	25.78	71.75	0	21.71	13.50	101.76	0
FR04	1.92	31.27	31.67	0	22.89	4.00	104.42	0
FR05	2.37	7.64	11.37	0	13.89	0.94	70.57	0
FR06	2.35	18.71	21.56	0	20.64	0.85	67.62	0
FR07	4.23	19.35	198.22	0	22.12	38.06	171.26	1.12
FR08	8.75	18.48	125.00	0	19.73	15.87	152.08	0
FR09	1.60	9.92	6.00	0	14.07	0	65.67	0
FR10	2.46	17.63	28.07	0	17.64	2.94	67.59	0
FR11	0.62	5.34	0.56	0	11.90	0	59.36	0
FR12	2.42	17.90	24.79	0	18.55	0.37	68.03	0
FR13	2.84	14.74	10.09	0	20.42	0	104.75	0
FR14	2.25	15.49	16.09	0	17.14	0	92.81	0
FR15	1.52	11.14	50.74	0	18.77	14.93	149.55	0
FR16	2.08	14.57	23.62	0	19.37	4.19	87.82	0
FR17	3.06	14.62	147.05	0	23.22	31.80	150.72	0.27
FR18	3.33	11.91	4.93	0	15.54	0	59.11	0
FR19	1.65	8.91	39.21	0	17.57	12.33	130.33	0.11
FR20	3.19	8.32	6.49	0	16.23	0	59.78	0
FR21	2.72	26.87	104.17	0	30.50	25.61	146.52	1.10
FR22	3.05	12.23	150.73	0	22.58	32.57	142.93	1.14

Table S2. Predicted concentrations of eight standard substances in 18 samples from Gaoma Erxi (μg/mL).

Samples from Gaoma Erxi.	predicted values (μg/mL)							
	GC	EGC	EGCG	GCG	EC	ECG	CAF	CG
GMEX01	2.00	18.54	23.80	0	19.10	0	62.96	0
GMEX02	0	33.19	12.80	0	23.68	0.20	75.09	0
GMEX03	1.37	20.01	29.93	0	18.74	5.26	94.88	0
GMEX04	0.68	20.34	14.35	0	18.16	1.49	81.89	0
GMEX05	5.42	17.79	30.09	0	19.08	0	99.91	0

GMEX06	0	2.53	0	0	10.46	0	1.78	0
GMEX07	2.14	31.81	29.79	0	22.13	3.04	70.74	0
GMEX08	0.18	3.08	0	0	11.74	0	57.34	0
GMEX09	2.10	12.89	10.13	0	15.10	2.55	46.04	0
GMEX10	1.45	24.91	26.93	0	24.81	2.13	88.20	0.81
GMEX11	2.44	50.29	68.95	0	46.86	16.26	136.88	3.68
GMEX12	2.31	14.78	22.66	0	21.77	1.83	106.34	0.69
GMEX13	2.13	20.06	26.67	0	24.95	0	76.83	0.08
GMEX14	0.52	2.77	0	0	13.96	0	137.10	0.09
GMEX15	3.14	21.88	55.78	0	31.09	10.16	106.73	0.61
GMEX16	0.28	26.34	49.36	0	30.83	12.27	173.40	2.19
GMEX17	1.64	15.24	22.34	0	27.01	2.38	83.85	0.94
GMEX18	8.74	54.91	202.86	0	56.85	46.80	160.28	7.18

Table S3. Predicted concentrations of eight standard substances in 23 samples from Yuntaishan (μg/mL).

**Samples from Yuntaishan.**	**predicted values (μg/mL)**							
	**GC**	**EGC**	**EGCG**	**GCG**	**EC**	**ECG**	**CAF**	**CG**
YTS01	1.88	10.30	107.20	0	20.69	21.11	127.43	0.48
YTS02	1.77	8.78	37.50	0	18.85	6.59	112.63	0.03
YTS03	2.50	15.94	157.04	0	23.22	38.56	128.28	1.69
YTS04	2.02	9.55	20.93	0	16.01	0	81.71	0
YTS05	1.64	13.17	32.33	0	21.62	11.10	111.40	0.13
YTS06	2.31	15.99	18.32	0	20.88	2.85	96.60	0
YTS07	1.78	6.84	38.73	0	17.12	7.74	110.49	0.53
YTS08	4.52	32.21	115.54	0	39.87	24.41	149.44	1.22
YTS09	2.73	3.66	50.37	0	16.19	11.55	145.89	0
YTS10	4.42	33.10	152.89	0	38.45	23.13	147.97	0.88
YTS11	1.32	7.42	2.27	0	16.83	0	52.85	0
YTS12	1.07	4.64	0.82	0	13.50	0	68.86	0
YTS13	4.08	24.40	139.54	0	28.24	22.37	143.89	1.43
YTS14	4.34	22.10	171.37	0	32.95	26.19	136.43	1.18
YTS15	1.17	9.15	14.29	0	19.04	0	88.76	0
YTS16	1.80	13.84	8.41	0	20.44	0	86.31	0
YTS17	3.45	35.07	76.80	0	33.74	8.47	156.41	1.64
YTS18	6.20	29.74	37.55	0	27.45	0	101.48	0.22
YTS19	1.61	11.92	20.34	0	17.92	0.92	82.99	0
YTS20	4.41	11.08	63.84	0	20.84	7.75	152.98	0.79
YTS21	3.22	33.45	27.88	0	32.11	0.76	174.18	0.78
YTS22	3.05	26.59	143.61	0	28.04	26.15	131.20	1.14
YTS23	3.76	26.63	51.93	0	30.48	8.55	101.63	0

Table S4. Orthogonal experimental design L9(3^4) for t-SNE hyperparameter optimization.

Case	Perplexity	Learning Rate	Iterations	Early Exaggeration	Silhouette Coefficient	Classification accuracy (%)
1	5	10	250	4	0.64	42.9
2	5	100	500	8	0.45	71.4
3	5	1000	1000	12	0.51	71.4
4	20	10	500	12	0.43	71.4
5	20	100	1000	4	0.51	71.4
6	20	1000	250	8	0.69	71.4
7	50	10	1000	8	0.49	71.4
8	50	100	250	12	0.82	85.7
9	50	1000	500	4	0.28	85.7

Optimal configuration (Case 8): Perplexity = 50, Learning Rate = 100, Iterations = 250, Early Exaggeration = 12.
